# Effect of 30 days of ketogenic Mediterranean diet with phytoextracts on athletes' gut microbiome composition

**DOI:** 10.3389/fnut.2022.979651

**Published:** 2022-10-25

**Authors:** Laura Mancin, Stefano Amatori, Massimiliano Caprio, Eleonora Sattin, Loris Bertoldi, Lorenzo Cenci, Davide Sisti, Antonino Bianco, Antonio Paoli

**Affiliations:** ^1^Department of Biomedical Sciences, University of Padua, Padua, Italy; ^2^Human Inspired Technology Research Center, University of Padua, Padua, Italy; ^3^Department of Biomolecular Sciences, University of Urbino Carlo Bo, Urbino, Italy; ^4^Department of Human Sciences and Promotion of the Quality of Life, San Raffaele Roma Open University, Rome, Italy; ^5^Laboratory of Cardiovascular Endocrinology, IRCCS San Raffaele Pisana, Rome, Italy; ^6^BMR Genomics srl, Padua, Italy; ^7^Sport and Exercise Sciences Research Unit, University of Palermo, Palermo, Italy; ^8^Research Center for High Performance Sport, UCAM, Catholic University of Murcia, Murcia, Spain

**Keywords:** ketogenic diet, gut microbiota, sport nutrition, exercise, athletes

## Abstract

**Background:**

Recent research suggest that gut microbiome may play a fundamental role in athlete's health and performance. Interestingly, nutrition can affect athletic performance by influencing the gut microbiome composition. Among different dietary patterns, ketogenic diet represents an efficient nutritional approach to get adequate body composition in athletes, however, some concerns have been raised about its potential detrimental effect on gut microbiome. To the best of our knowledge, only one study investigated the effect of ketogenic diet on the gut microbiome in athletes (elite race walkers), whilst no studies are available in a model of mixed endurance/power sport such as soccer. This study aimed to investigate the influence of a ketogenic Mediterranean diet with phytoextracts (KEMEPHY) diet on gut microbiome composition in a cohort of semi-professional soccer players.

**Methods:**

16 male soccer players were randomly assigned to KEMEPHY diet (KDP *n* = 8) or western diet (WD *n* = 8). Body composition, performance measurements and gut microbiome composition were measured before and after 30 days of intervention by 16S rRNA amplicon sequencing. Alpha-diversity measures and PERMANOVA was used to investigate pre-post differences in the relative abundance of all taxonomic levels (from phylum to genus) and Spearman's correlations was used to investigate associations between microbial composition and macronutrient intake. Linear discriminant analysis was also performed at the different taxonomic levels on the post-intervention data.

**Results:**

No differences were found between pre and post- dietary intervention for microbial community diversity: no significant effects of time (*p* = 0.056, ES = 0.486 and *p* = 0.129, ES = 0.388, respectively for OTUs number and Shannon's ENS), group (*p* = 0.317, ES = 0.180 and *p* = 0.809, ES = 0.047) or time × group (*p* = 0.999, ES = 0.01 and *p* = 0.230, ES = 0.315). *Post-hoc* paired Wilcoxon test showed a significant time × group effect for *Actinobacteriota* (*p* = 0.021, ES = 0.578), which increased in the WD group (median pre: 1.7%; median post: 2.3%) and decreased in the KEMEPHY group (median pre: 4.3%; median post: 1.7%). At genus level, the linear discriminant analysis in the post intervention differentiated the two groups for *Bifidobacterium* genus (pertaining to the *Actinobacteria* phylum), *Butyricicoccus* and *Acidaminococcus* genera, all more abundant in the WD group, and for *Clostridia UCG-014* (order, family, and genus), *Butyricimonas, Odoribacterter* genera (pertaining to the Marinifilaceae family), and *Ruminococcus* genus, all more abundant in the KEMEPHY group.

**Conclusions:**

Our results demonstrate that 30 days of KEMEPHY intervention, in contrast with previous research on ketogenic diet and gut microbiome, do not modify the overall composition of gut microbiome in a cohort of athletes. KEMEPHY dietary pattern may represent an alternative and safety tool for maintaining and/or regulating the composition of gut microbiome in athletes practicing regular exercise. Due to the fact that not all ketogenic diets are equal, we hypothesized that each version of ketogenic diet, with different kind of nutrients or macronutrients partitioning, may differently affect the human gut microbiome.

## Introduction

The human intestinal tract is composed of a considerable population of microorganisms (microbiota) and its corresponding gene complement (microbiome), that symbiotically live within the host. In recent years, the awareness of the importance of microbial community in human health has increased tremendously, making the science of microbiome a key area for life sciences (1). Intrinsic and extrinsic factors including age, environment, birth delivery route, breastfeeding, antibiotics, genetic background, human leukocyte antigen, dietary factors, and exercise, impact the microbial composition and function, with the diet and exercise act as primary modulators (2–7). More specifically, in sport nutrition, diet represents one of the most important tools that athletes use to optimize their fitness, performance and recovery and macro nutrients manipulation are often adopted to optimize training outcomes and competitions' performance. For example, carbohydrates represent a primary fuel source during physical activity, and they are fundamental to maintain and refill athlete's muscle glycogen stores. To date, recent evidence suggests that carbohydrates may influence athletic performance also *via* the modulation of gut microbiome (8). Indeed, the effect of carbohydrates on the gut microbiome differs widely as a function of microbiota-accessible carbohydrates (MACs) commonly referred to as dietary fiber, content, and types. Dietary MACs are found in a variety of sources including plants, animal tissue, or food-borne microbes and represent the source of carbohydrates that are metabolically available for gut microbes. MACs hold a role of “primary fermenters” within the colonic ecosystem and generally tend to increase the production of the beneficial short chain fatty acids (SCFAs) producing bacteria such as *Bacteroides, Firmicutes* and *Actinobacteria* (9). Differently, an increased consumption of protein among athletes, may lead to an excessive protein fermentation associated with the increased abundance of related taxa such as *Clostridium* and *Proteobacteria*. In sport nutrition, an additional area of interest is also represented by the study of ketogenic diet (KD) effects on athletes' health and performance. Indeed, high fat-low carbohydrate diet, such as ketogenic diet, has gained popularity among athletes and practitioners for its potential application in sports (10). KD represents a dietary protocol consisting of high-fat, adequate protein and < 20 g of carbohydrate daily (or 5% of total daily energy) (10). This nutritional approach has been used since the 1920 as a treatment for refractory epilepsy (11) and it has gained popularity as a potential treatment for obesity and related metabolic disorders (12). Indeed, increased amount of evidence point out that KD may represent an efficient and safe solution to get adequate body composition and maintain a general good health. The metabolic shift induced by ketogenic diet and some of the complex metabolic pathways involved in “ketotic state” has suggested a possible use of ketogenic diet in sports (10). For example, the use of KD may represent a safe strategy for the athletes who need to reduce body weight and body fat while maintaining lean mass and performance (13). One of the concerns raised about the use of KD for sport purposes is related to its putative negative impact on gut microbiome (14). On the other side, substantial changes in microbiome composition have been also attributed to exercise. To date, some studies reveal that exercise may increase the gut microbiota diversity and associated microbial-derived metabolites (2, 15). Observational studies have revealed that high-level athletes have an increased microbial α-diversity (a measure of microbiome diversity of a single sample), lower inflammatory markers and a higher microbial production of short chain fatty acids (SCFAs) (15). For example, Clarke et al., compared the gut microbiota of professional Irish male rugby players with two groups of healthy, non-athletes subjects matched for body mass index (BMI): (>28 kg/m^2^) and (< 25 kg/m^2^) and found that the microbial diversity of rugby players was higher compared with both non-athletes groups (2). More recently, Scheinman et al. collected and sequenced the stool samples from a cohort of athletes participating to the Boston Marathon (1 week before and 1 week after), along with a group of healthy-non athletes' controls. The researchers found that the most differentially abundant specie was *Veillonella atypica*, a Gram-negative bacterium that metabolize lactate into acetate and propionate *via* the methylmalonyl-CoA pathway. Further, compared with mice gavaged with *Lactobacillus*, the transplantation of stool containing the *Veillonella* significantly improved submaximal treadmill run time to exhaustion, suggesting a potential role for *Veillonella atypica* in improving athletic performance. The authors suggested the possibility that the lactate produced during sustained exercise could be converted by *Veillonella atypica* into propionate, identifying a new microbiota-driven enzymatic process that may improve athletic performance (6). To the best of our knowledge, only one study investigated the effect of KD on the gut microbiota in athletes (a cohort of elite race walkers) (16), while no studies are available in a model of mixed endurance/power sport such as soccer. In our recent article (13) we investigated the effect of 30 days of ketogenic diet on body composition, muscle strength, muscle area and metabolism in a cohort of semi-professional soccer players. The athletes who underwent the KD intervention lost body fat mass without detrimental effect on strength, muscle mass and power. However, considered the suggested detrimental effect of KD on gut microbiome (16), the aim of the current study was to assess the gut microbiome composition of semi-professional soccer players who participated in the above cited study, to understand whether and how the gut microbiota changes in response to thirty-days of ketogenic Mediterranean diet with phytoextracts (KEMEPHY) diet.

## Materials and methods

### Participants

This is a secondary analysis of a previous published research (13).

A more detailed description of the experimental study and physiological measures can be found (13). Sixteen semi-professional soccer players (25.5 ± 2.8 years, 77.2 ± 11.88 kg) were recruited for the study. The exclusion criteria were: participants with a body fat percentage over 32%, (determined *via* dual energy X-ray absorptiometry DXA), cardiovascular, respiratory, gastrointestinal, thyroid or any other metabolic diseases, weight change ± 2 Kg over the last month, adherence to special diets, use of nutritional supplements (except a daily multivitamin-mineral), use of antibiotics (17), use of medication to control blood lipids or glucose. The anthropometric details of the subjects enrolled in the study were provided in our previous published study (13).

During the study players were asked to keep their normal training schedule (8 h of training/week). After the medical health screening, all the subjects read and signed the informed consent with the description of the testing procedures approved by the Ethical Committee of the Department of Biomedical Sciences, University of Padua, and conformed to standards for the use of human subjects in research as outlined in the Declaration of Helsinki, Clinical Trial registration number NCT04078971.

### Study design and procedures

The study was a randomized, parallel arm, controlled, prospective study in which gut microbiota was tested before and after 30 days of KEMEPHY protocol. Subjects undergone to several anthropometric and performance measurements described in our previous paper (13).

Subject were randomly assigned to the KEMEPHY diet (KDP *n* = 8) group or Western Diet (WD *n* = 8) group, through an on-line random number calculator (https://www.graphpad.com/quickcalcs/randMenu/), matched for percentage of body fat.

**Figure 1:**
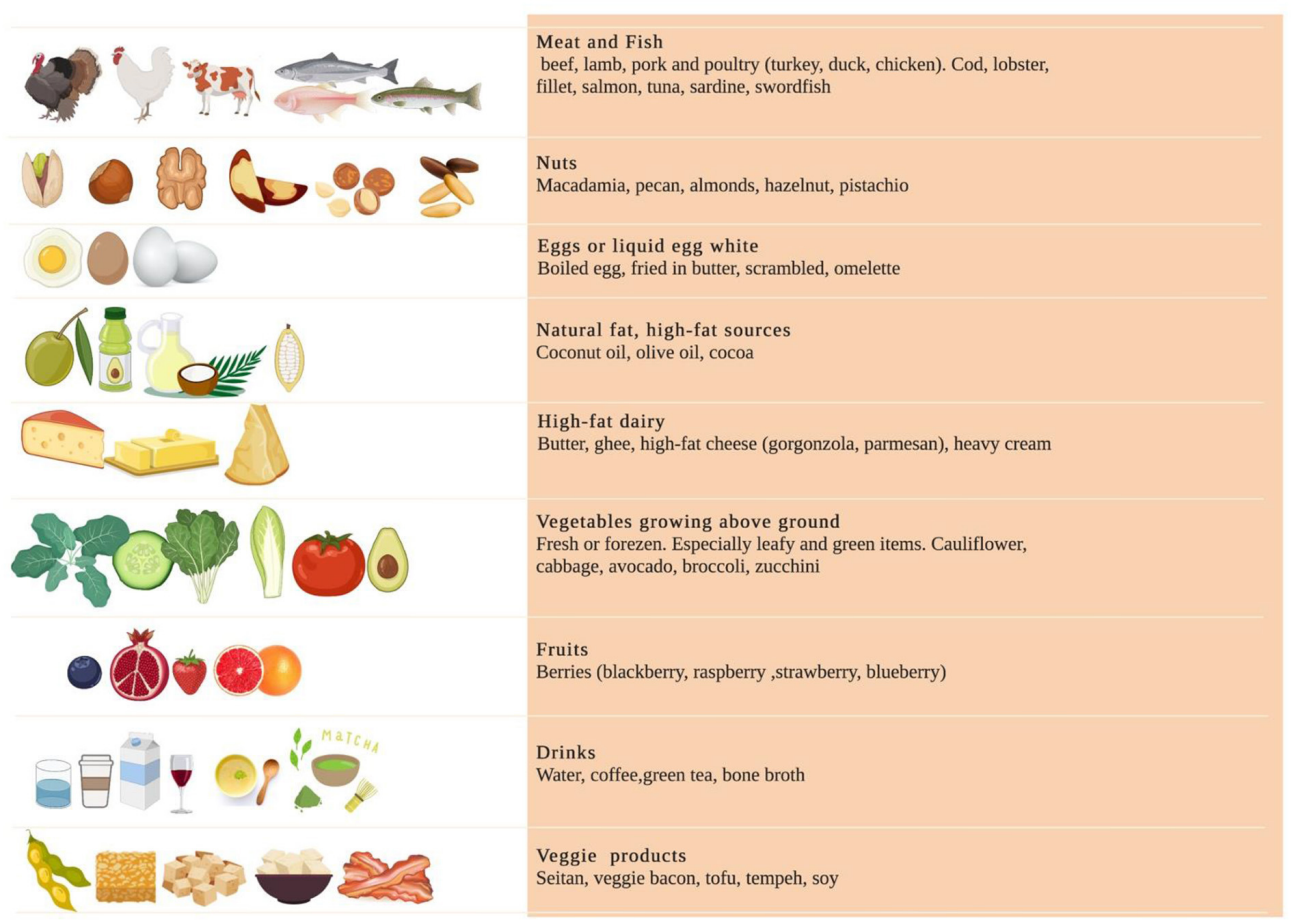
Detailed list of food provided.

The workload of all athletes was over-imposable because the coach and trainers strictly controlled the training schedule, and they were instructed to maintain the same level of physical activity throughout the study (The study protocol is shown in detail in our previous article, Figure 1).

### Dietary intervention

Before the start of the study, athletes were provided nutritional counseling and resources to better adhere to KEMEPHY. Resources included food lists containing the food prohibited and permitted in ketogenic diet and electronic-suggested daily meal plans, meal recipes. The food lists encouraged on eating unprocessed meat including beef, veal, poultry; fish such as eel, mackerel, salmon, sardines; raw and cooked vegetables, cold cuts such as dried beef, eggs and seasoned cheese (parmesan); Konjac; fruits with the lowest glycemic index (blueberry, raspberry), raw nuts and seeds, ghee butter, butter, plant oils and fats from avocado, coconut and green olives (18). A detailed list is provided in Figure 1, [modified from Antonio Paoli et al. (13)].

The drinks permitted were tea, coffee, herbal extracts without sugar and it was allowed a “*Keto cocktail*” once a week, made up of gin and soda. Moreover, since the nutritional protocol of KD it may be hard to be maintained for long periods due to the lack of sweet taste (19), many ready-to-eat ketogenic products (RKP) have been provided in addition to usual low carbohydrate foods (20). The present study indeed tested some ready-to-eat foods selected from the product range of Tisanoreica^®^ snacks and meals (Gianluca Mech S.p.A., Asigliano Veneto, Vicenza, Italy) and Le Gamberi Food^®^ and meals.

In our protocol we used some RKP as a ketogenic pasta (selected with a ketogenic ratio of fats: protein+carbohydrate equal to 4:1) (Le Gamberi Foods, Forlì, Italy), and other RKP (specialty meals and drinks) that mimics the taste of carbohydrates, constituted principally of high-quality protein (18 g of protein per portion), fibers, and electrolytes (mainly magnesium and potassium) (Tisanoreica^®^ by Gianluca Mech S.p.A., Asigliano Veneto, Vicenza, Italy), detailed in Table 1. Among the products selected, there were 4 sweets RKP products: chocolate biscuits CB (Cioco-Mech); chocolate and hazelnut balls CHB (Bon Mech); apple-cinnamon biscuits ACB (T-Biscuit); chocolate-almonds-pistachio bar CAPB (T-Smart) and one savory product: pasta P1 (Le Gamberi Pasta).

**Table 1:** Plant extracts and composition.

**Extracts 1, 30ml/day**
***Durvillea antarctica***, black radish, mint, liquorice, artichoke, horsetail, burdock, dandelion, rhubarb, gentian, lemon balm, chinaroot, juniper, spear grass, elder, fucus, anise, parsley, bearberry, horehound
**Extracts 2, 30ml/day**
Horsetail, asparagus, birch, cypress, couch grass, corn, dandelion, grape, fennel, elder, rosehip, anise
**Extracts 3, 30ml/day**
Eleuthero, ***Eurycoma longifolia***, ginseng, corn, ***Miura puama***, grape, guaranà, arabic coffee, ginger
**Extracts 4, 30ml/day**
***Linum usitatissimum*** L., ***Gelidium amansii, Rheum officinalis*** L., ***Cynara scolymus*** L., ***Matricaria chamomilla*** L., ***Gentiana lutea*** L., ***Mentha piperita*** L., ***Pimpinella anisum*** L., ***Glycyrrhiza glabra*** L., ***Raphanus sativus*** L., ***Foeniculum vulgare Mill., Althaea officinalis*** L., ***Melissa officinalis*** L., ***Juniperus communis L***.

Both diets were designed to be isoproteic i.e., same amount of protein (1.8 g × Kg^−1^ × body weight^−1^ × day ^−1^). The distribution of macronutrients during the KEMEPHY was carbohydrate (< 30 g × day^−1^; < 10%) protein 1.8g × Kg^−1^ × body weight^−1^ × day ^−1^ (~25–30%), fats *ad libitum*. Moreover, each subject was provided of three herbal extracts [Table 1, Antonio Paoli et al. (13)] according to commercial ketogenic protocol (Tisanoreica^^®^^, Gianluca Mech S.p.A., Asigliano Veneto, Vicenza, Italy).

During the first week, subjects were provided of pure medium chain triglyceride oil (MCT oil: 20 g Named^^®^^ Natural Medicine), in order to facilitate ketosis (21) and to allow players maintaining the same work load during training sessions. WD group was provided of a diet similar to western diet, thus the intake of protein has been increased to 1.8 g × Kg^−1^ × body weight^−1^ × day^−1^ in order to be make the two diets isoproteic. The WD was composed mainly of whole cereals (spelt, rye, oat) and pseudo-cereals (buckwheat, quinoa, amaranth), whole grain pasta, potatoes, meet, fish, vegetables, fruit, legumes, olive oil, milk, and red wine (at most 1 glass per day). Thus, the WD ensured a constant energy and macronutrient balance: protein 1.8 g × Kg^−1^ × body weight^−1^ × day^−1^, (~ 30%), fats ~20–25% and carbohydrate ~50–55%. WD diet was also designed to contain < 10% saturated fat and < 300 mg cholesterol/day.

It should be stressed that, as it can be noted, the WD diet we provided to the athletes was totally different from the typical high-fat, high sucrose Western diet usually adopted in research studies.

In both groups protein intake was distributed equally throughout the day (every 3–4 h) and pre-sleep casein protein intake (30–40 g) was provided in both group after training evening session, as indicated by the ISSN's position stand (22). The diets were explained to all subjects during an individual visit and dietary intake was measured by validated 3-food-diary that has been used in the past in studies with athletes (23) and analyzed by *Nutritionist Pro*™ *(AxxyA systems, Arlington, VA)*.

Subjects received the specific instruction for completing detailed weighed food records during 7 day-periods for each diet and were daily monitored by call interviews each day after dinner. To ensure that carbohydrates were restricted throughout the KEMEPHY diet, subjects tested their urine daily using reagent strips at the same time of the day (Ketostix semiquantitative urine strips, Bayer, Leverkusen, Germany), recording the result on log sheet and, once or twice a week, subjects were tested by *GlucoMen LX Plus (Menarini Diagnostics, Firenze, Italy)* to detect ketones concentration in capillary blood. Subjects received follow-up counseling and dietetic education if necessary. Additionally, a *WhatsApp* (Meta Inc., Mountain View, CA, USA) group was created and some applications for smartphone were provided (*Keto-diet tracker,* https://ke.to/; Keto-app, https://ketodietapp.com/), to track their food daily intake.

### Feces sampling and DNA extraction

Feces samples were collected at baseline and after 30 days of dietary protocol.

100–150 mg of feces were collected using sterile swab (FLmedical, Italy) tubes (Starlab Group, Italy) and preservative buffer (Zymo Research, USA) in the morning of the day of starting KEMEPHY and after thirthy days. Samples were sent to BMR Genomics srl (*via* Redipuglia, 22, 35131 Padova, PD) within 2 days and stored at −20 ^°^C until DNA extraction. DNA was extracted using Cador Pathogen 96 QIAcube HT Kit (Qiagen srl, DE) with lysis step modification according to Mobio PowerFecal kit (Qiagen srl, DE).

### 16S rRNA gene sequence data processing and analysis

The V3-V4 regions of the 16S ribosomal RNA gene were amplified using Illumina tailed primers Pro341F (5^′^-TCGTCGGCAGCGTCAGATGTGTATAAGAGACAG-CCTACGGGAGGCAGCA-3^′^) and Pro805R (5^′^-GTCTCGTGGGCTCGGAGATGTGTATAAGAGACAGGACTACNVGGGTATCTAATCC-3^′^) using Platinum Taq (Thermo Fisher Scientific Inc, USA) by means PCR (94^°^C for 1 min, followed by 25 cycles at 94^°^C for 30 s, 55^°^C for 30 s, and 68^°^C for 45 s, and a final extension at 68^°^C for 7 min). PCR amplicons were purified by means Agencourt AMPure XP Beads 0.8X (Beckman Coulter, Inc., CA, USA) and amplified following the Nextera XT Index protocol (Illumina, Inc., CA, USA). The indexed amplicons were normalized by SequalPrepTM Normalization Plate Kit (Thermo Fisher Scientific Inc.) and multiplexed. The pool was purified with 1X Magnetic Beads Agencourt XP (Beckman Coulter, Inc.), loaded on the MiSeq System (Illumina, Inc.) and sequenced following the V3-−300PE strategy. The bioinformatic analysis was performed by means QIIME 2 2021.4 version (24). Raw reads were firstly trimmed applying Cutadapt to remove residual primer sequences and then processed with DADA2 plug-in (25) to perform the denoising step. DADA2 was run with default parameters except for the truncation length: forward and reverse reads were truncated at 260 and 245 nucleotides, respectively. The resulting Amplicon Sequence Variant (ASV) sequences were filtered out by applying a 0.01% frequency threshold in order to discard singletons and very rare sequences. All the samples included in the analysis was rarefied. The value of rarefaction is 32,232 reads (Supplementary Figure 1).

The more recent available Silva 138 database (26) as used to associate the taxonomy to the remaining ASVs for the final analysis; moreover we earlier performed also an analysis with Green genes v.13-8 database that will be briefly discussed to better understand the variability due to the database utilized.

### Statistical analysis

Results are presented as mean and standard deviation (SD), or median and quartiles (Q1-Q3) where appropriate. Alpha diversity indexes (OTUs number and Shannon's Effective Number of Species) were computed with the diversity function of the *vegan* R package, and time, group and time × group effects were tested using a Wilcoxon test for paired data (interaction effect was checked while performing the test on delta values); a false discover rate (FDR) with Benjamini-Hochberg correction was applied to account for multiple testing. Effect sizes were calculated with the *rstatix* and *coin* R packages. Common interpretations of Wilcoxon effect sizes (r) are: 0.10–0.3 (small effect), 0.30–0.5 (moderate effect) and ≥0.5 (large effect). A dissimilarity matrix with Bray-Curtis distance was calculated, and a Permutational Analysis of Variance (PERMANOVA) for repeated measures was used to test pre-post differences between the two groups (KDP vs. WD) in the relative abundances at phylum and genera taxonomic levels, using the *adonis* R function, and *post-hoc* comparisons were performed with a paired Wilcoxon test with FDR correction. Furthermore, after ruling out baseline differences in the microbial composition at baseline, data were filtered for the presence of each taxon in at least 70% of the subjects, and a linear discriminant analysis (LDA) was performed at the different taxonomic levels (from phylum to genus) on the post-intervention data (LEfSe; LDA Score >2.0, *p* < 0.05); significant different taxa were graphically represented on a cladogram. To assess correlations between macronutrient intake (7-days food diary) and pre-post treatment variations in body composition, fitness measures and genera abundances, a Spearman correlation matrix was computed: significant correlations were extracted (Spearman *r*_**0.05, 14**_ ≥ 0.503), and represented in a circular plot using the *circlize* R package. Analyses were performed using R Studio 4.1.1; the significance level was fixed at the standard value of 0.05.

## Results

### Dietary nutrition intake

There were no differences in dietary nutrient intakes between groups at baseline. Subjects adhered to the given instructions for both diet interventions according to analysis of diets records (3 days food-diary before the study and 7 days food-diary during the study). During the diet interventions, all dietary nutrients were significantly different between the KEMEPHY and WD diets. Indeed, the intake of CHO g/day and % in KEMEPHY and WD group was, respectively (KDP = 22 ± 5 g/day; WD = 220 ± 56 g/day, *p* < 0.0001), (KDP = 9 ± 3 %; WD = 51 ± %, *p* < 0.0001) while the intake of % fat was (KDP = 64 ± 3%; WD = 20 ± 8 %; *p* < 0.0001). In addition, the total energy intake was reduced during both the treatments but without a significant difference between groups (KDP = 1.984 ± 340Kcal/day; WD = 1.752 ± 320Kcal/day), (*p* > 0.05). The complete results about dietary nutrition intake during the intervention are shown in Table 3 of the previous study (13). For an easier understanding we reported pre- and post- daily dietary energy and nutrient intake in brief in Table 2.

**Table 2:** Daily dietary energy and nutrient intake at baseline and during KEMEPHY diet (KDP) and Western Diet (WD).

	**KDP Pre**	**KDP Post**	**WD Pre**	**WD Post**	Time*Diet effect (p)
Total (Kcal/die)	2356 ± 450	1984 ± 340	2146 ± 230	1752 ± 320	n.s.
Carbohydrates (g/die)	350 ± 66	22 ± 5	363 ± 34	220 ± 56	*p < * 0.05
Protein (g/die)	105 ± 20	130 ± 25	121 ± 23	129 ± 28	n.s.
Fat (g/die)	107 ± 20	132 ± 27	110 ± 16	38 ± 10	n.s
Carbohydrates (%)	49 ± 6	9 ± 3	51 ± 4	51 ± 4	*p < * 0.05
Protein (%)	15 ± 3	28 ± 4	14 ± 6	28 ± 3	n.s.
Fat (%)	35 ± 4	64 ± 3	33 ± 2	20 ± 8	*p < * 0.05
Protein (g/Kg bw)	1.37 ± 0.5	1.85 ± 0.3	1.59 ± 0.4	1.83 ± 0.2	n.s.
Saturated Fat (g)	35 ± 10	45 ± 12	36 ± 4	15 ± 3	*p < * 0.05
Monounsaturated fat (g)	28 ± 6	49 ± 16	27 ± 5	9 ± 5	*p < * 0.05
Polyunsaturated fat (g)	16 ± 3	21 ± 5	16 ± 9	5 ± 2	*p < * 0.05
Cholesterol (mg)	304 ± 101	720 ± 187	303 ± 98	167 ± 65	*p < * 0.05
Fiber (g)	13 ± 2	10 ± 3	11 ± 9	15 ± 4	n.s

Values are mean ± SD, Analysis performed on 3 days of diet records during habitual diet and 7 days during KDP and WD. n.s., not significant.

### Microbiota composition

**Figure 2:**
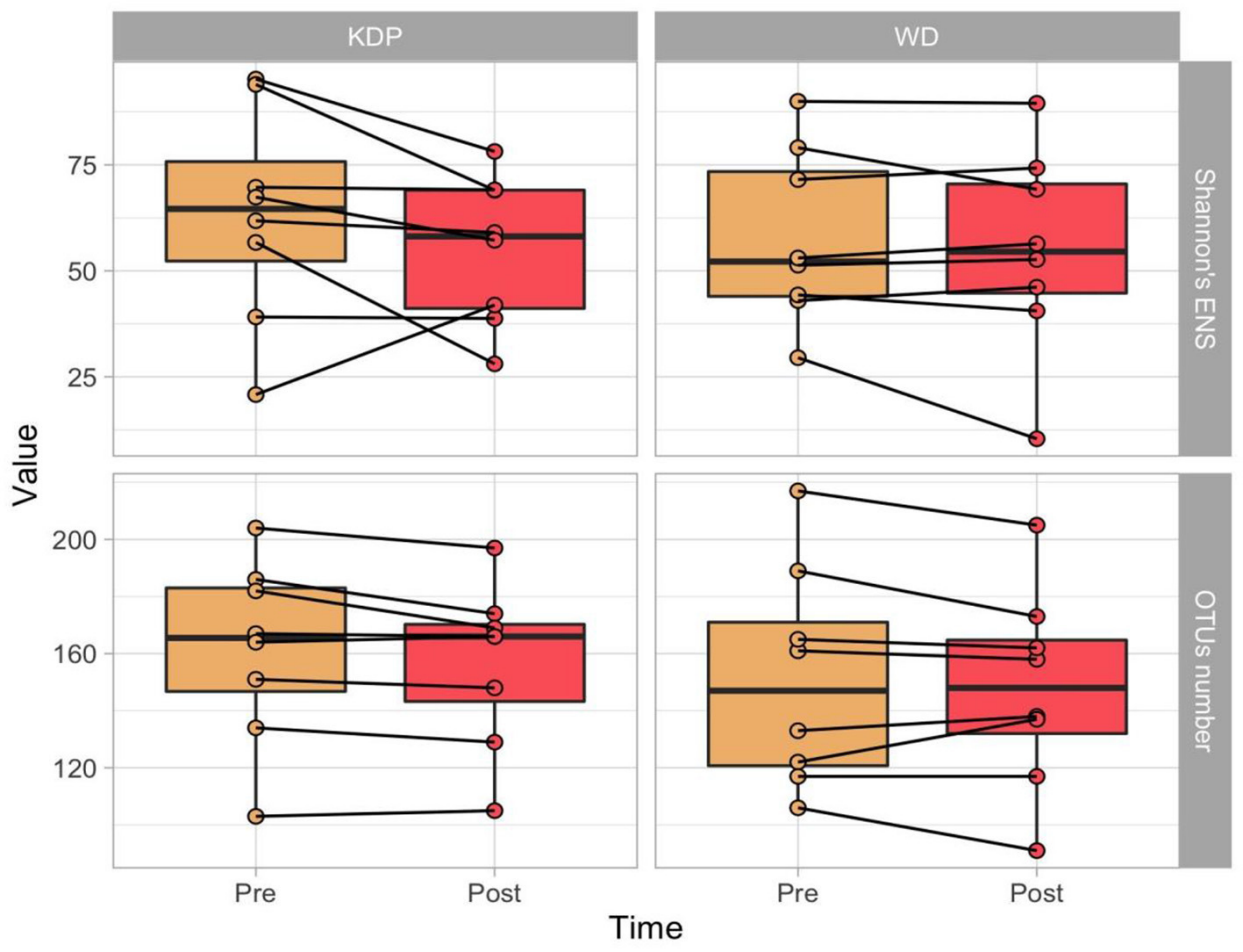
Paired boxplots of OTU's number and Shannon's Effective Number of Species (ENS) in the two groups (KDP vs. WD), at the two time points (Pre and Post Intervention).

As alpha diversity measures, the OTUs number and the Shannon's Effective Number of Species (ENS) were calculated. No significant effects of time (*p* = 0.056, ES = 0.486 and *p* = 0.129, ES = 0.388, respectively for OTUs number and Shannon's ENS), group (*p* = 0.317, ES = 0.180 and *p* = 0.809, ES = 0.047) or time × group (*p* = 0.999, ES = 0.01 and *p* = 0.230, ES = 0.315) were found (Figure 2).

**Figure 3:**
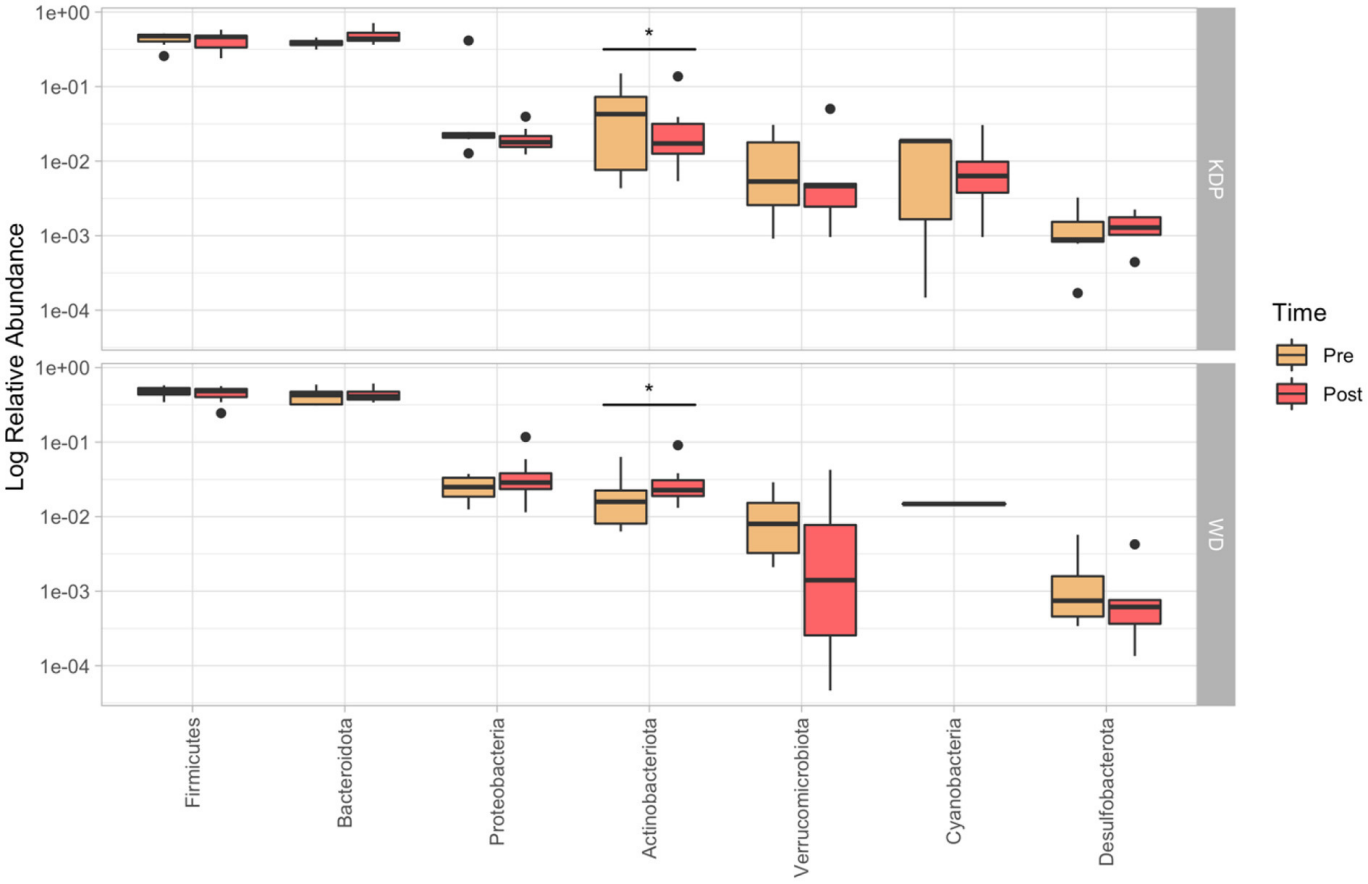
Relative abundance (in log_10_ scale) of the more represented phyla (>0.1%) in the pre- and post-intervention, for KDP and WD groups. Stars represent a significant time × group interaction (*p* < 0.05).

PERMANOVA for paired data did not find any significant time × group interaction effect for none of the analyzed taxonomic levels (*p* > 0.05). Nonetheless, *post-hoc* paired Wilcoxon test showed a significant time × group effect for *Actinobacteriota* (*p* = 0.021, ES = 0.578), which increased in the WD group (median pre: 1.7%; median post: 2.3%) and decreased in the KDP group (median pre: 4.3%; median post: 1.7%) (Figure 3).

*Firmicutes/Bacteroidetes* ratio was 1.11 (1.07–1.23) in pre and 0.99 (0.73–1.15) in post, and 1.07 (0.99–1.67) in pre and 1.16 (0.94–1.23) in post conditions, in KDP and WD groups, respectively. No significant effect was found for the time × group interaction (*p* > 0.05).

**Figure 4:**
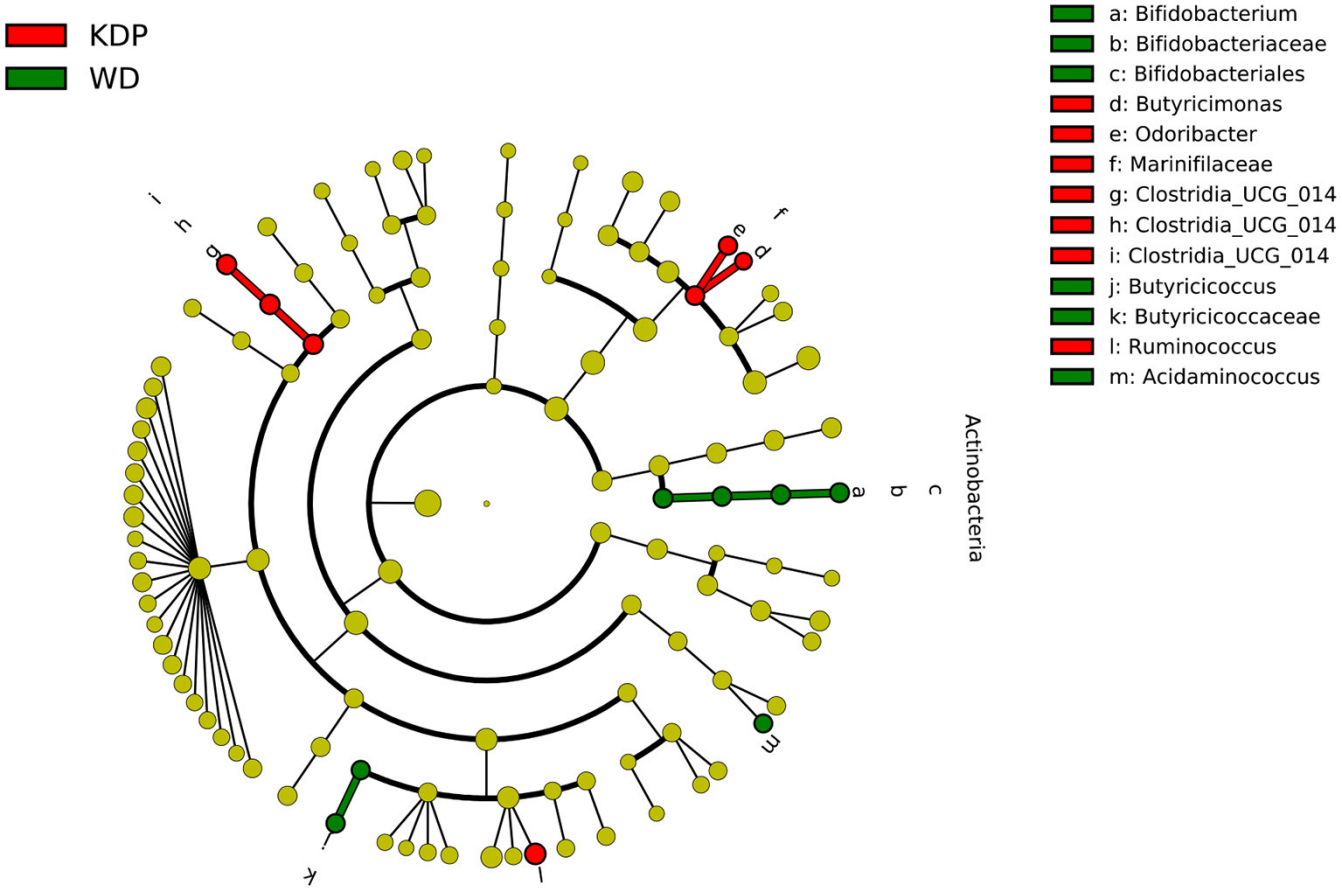
Differential taxa between the KDP and WD groups in the post-intervention (LEfSe analysis, adjusted *p* < 0.05, log 2 fold change >2).

The linear discriminant analysis in the post intervention differentiated the two groups for *Bifidobacterium* genus (pertaining to the *Actinobacteria* phylum), *Butyricicoccus* and *Acidaminococcus* genera, all more abundant in the WD group, and for *Clostridia UCG-014* (order, family, and genus), *Butyricimonas* and *Odoribacterter* genera (pertaining to the *Marinifilaceae* family), and *Ruminococcus* genus, all more abundant in the KDP group (Figure 4).

**Figure 5:**
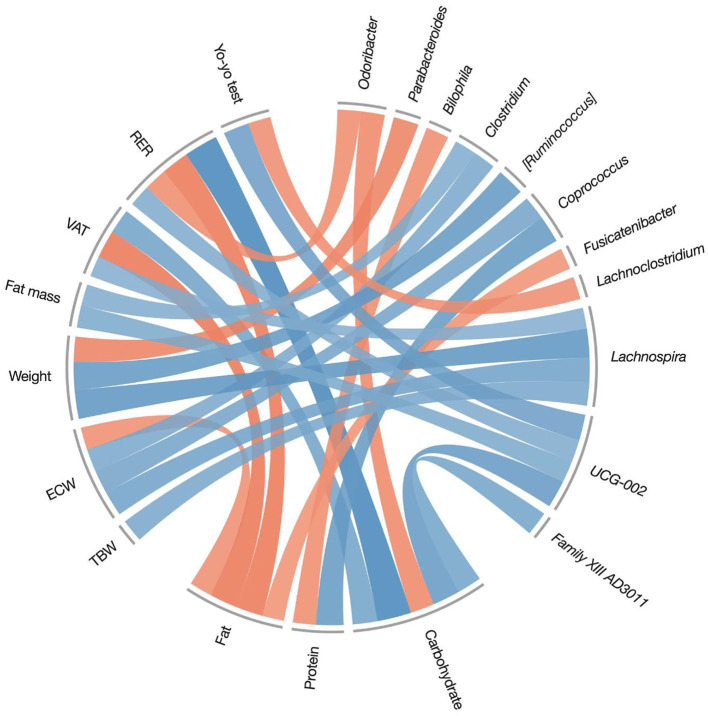
Spearman's correlations between macronutrient intake during the treatment period (7 days food-diary), and post-pre variations on body composition measures, fitness measures, and genera relative abundances. Only significant correlations were reported (*r*_0.05, 14_ ≥ 0.503). Positive correlations are represented by blue color and negative correlations by red color. TBW, total body water; ECW, extracellular water; VAT, visceral adipose tissue; RER, respiratory exchange ratio.

To investigate the associations between the macronutrient's intake during the intervention and the variations in genera abundances and environmental variables (i.e., anthropometric and performance measures), genera were filtered taking into consideration only those which were present in at least 70% of the subjects, both in pre- and post-interventions. Spearman's correlations were then calculated, and after applying a filter to those statistically significant (*r*_0.05, 14_ ≥ 0.503), were reported on a circle plot (Figure 5). For an easier interpretation of the correlations presented in Figure 4, pre- and post-treatment variations of anthropometric and performance measures are reported in brief in Table 3 (for the full table of results please see reference Table 4 in our previous study) (13).

**Table 3:** Anthropometric and performance variables pre- and post-intervention [modified from Antonio Paoli et al. (13)].

	**KDP Pre**	**KDP Post**	**WD Pre**	**WD Post**	**Time × Group effect (p)**
Body weight (kg)	78.2 ± 11.7	73.9 ± 9.4	76.2 ± 12.0	73.8 ± 10.1	n.s.
Fat mass (kg)	19.47 ± 4.07	17.92 ± 3.81	18.88 ± 6.67	17.96 ± 6.30	0.036
VAT (g)	388 ± 66	325 ± 54	355 ± 104	328 ± 101	0.0018
ECW (L)	19.93 ± 3.39	18.99 ± 2.63	19.75 ± 2.96	19.58 ± 2.97	n.s.
TBW (L)	49.79 ± 6.43	48.80 ± 5.39	48.84 ± 6.55	48.31 ± 6.47	n.s.
RER	0.87 ± 0.09	0.75 ± 0.04	0.86 ± 0.05	0.83 ± 0.04	0.0008
REE (kcal/Kg bw/day)	23.4 ± 0.8	23.3 ± 0.8	22.3 ± 1.0	22.4 ± 0.8	n.s.
Yo-yo test (m)	880.4 ± 244	1123 ± 266	683 ± 388	911 ± 378	n.s.

VAT, visceral adipose tissue; ECW, extracellular water; ICW, intracellular water; TBW, total body water; CSA, cross-sectional area; RER, respiratory exchange ratio; REE, resting energy expenditure; n.s., not significant.

In Figure 4, blue color represents positive correlations while red represents negative ones; the color intensity represents the strength of the correlation. Carbohydrate intake was strongly (*r* = 0.84) associated with a modification in the respiratory exchange ratio (RER), confirming the result in Table 3, which showed a significant reduction of RER in the KDP group. In other words, players in the KDP group that had less carbohydrate in their diet showed a greater decrease in RER, a sign of an increased reliance on oxidative metabolism. In addition, carbohydrate intake was inversely correlated with changes of *Odoribacter* genus abundance (*r* = −0.59), the latter being also negatively associated to changes in RER (*r* = −0.57). This association is coherent with the significant time × group effect in RER presented in Table 3, as *Odoribacter* genus were found to be more abundant in the KDP group (Figure 3). Fat intake, in contrast, was negatively associated with variations of RER (*r* = −0.68), visceral adipose tissue (VAT) (*r* = −0.69), extracellular water (ECW) (*r* = −0.55) and *Fusicatenibacter* genus (*r* = −0.53). Reductions in weight were associated with a reduced abundance of *Ruminococcus torques* (*r* = 0.68) and *Lachnospira* (*r* = 0.71) genera, and inversely correlated with *Parabacteroides* genus abundance (*r* = −0.62).

## Discussion

The human gut microbiome is well recognized to be implicated in the promotion-maintenance of health as well in some disease states (27).

Given its plasticity, the gut microbial community can be affected by several factors including genetics, nutrition, environment, exercise and exposure to antibiotics; however, among these contributors, diet elicits the predominant influencing factor (28). To date, while only one study investigated the effect of ketogenic diet in sport's performance and gut microbiome in endurance discipline (16), no data are available about the effect of ketogenic diet on gut microbiome composition and athlete's performance in team sport.

In this study we demonstrate that 30 days of KEMEPHY did not affect the overall gut microbiome of athletes in terms of alpha- diversity indices (the total number of species and the Shannon's Effective Number of Species); however, both groups presented a significant variation both at phylum and genus levels composition (Figure 1).

Indeed, the phylum of *Actinobacteria* was significantly decreased in the KEMEPHY and increased in the WD group (Figure 3), while *Clostridia UCG-014, Butyricimonas, Odoribacterter and Ruminococcus* genera were significantly increased after KDP intervention (Figure 4).

Although our data are in contrast with previous studies identifying a positive association between “high fat diet” and impairment on gut microbiome (14, 34, 35), our results are not surprising since the previous studies investigated the effect of a high-fat, high sugar, Western diet on gut microbiome and did not investigate the effect of ketogenic diet (14, 34, 36) that represent a unique, specific dietary pattern.

In addition, many studies (29–32) investigating the effect of a high-fat diet on gut microbiome tested only mouse models fed a refined high-fat, low fiber diet with animals fed a standard chow diet, high in soluble fibers. For this reason, the conclusions arising from animal studies cannot be adopted to predict the outcomes of a ketogenic diet and, consequently, its associated effect on human gut microbiome (33).

As a matter of fact, in humans, Turnbaugh et al. recently confirmed (34) that ketogenic diets differentially alter the composition of gut microbiome when compared to high-fat diet and, further, the authors showed that only ketogenic diet was able to provide positive gut-associated systemic outcomes (34).

Moreover, another explanation for the maintenance of microbial diversity after KEMEPHY intervention may rely on the specific composition of our KEMEPHY diet. Indeed, when investigating the effect of a ketogenic diet on gut microbiome and health parameters, it should be considered not only the amount of fat (i.e., 70–80% fat from total daily calories), but also the different type and quality of fats. Different types of fat are associated with different effects on the gut microbiome and, consequently, with different effects on intestinal and systemic health (35–37). If on one side saturated fats are associated with decreased microbiome diversity (14) in humans, polyunsaturated fat such as omega-3 did not affect microbial diversity and richness. Polyunsaturated fats have the capacity to improve gut epithelial integrity and gastrointestinal health through their ability to produce SCFAs (38). In our study, the KEMEPHY diet was highly composed in mono-polyunsaturated fat (49 ± 16 g and 21 ± 5 g, respectively) differently from the WD diet which was lower (9 ± 5 g and 5 ± 2, respectively) (13). We hypothesized that sources of omega-3 fatty acids may have act synergically with ketone bodies to promote an anti-inflammatory state (39), also influencing the intestinal microbiome by increasing the production of SCFAs (37). However, further studies investingating the hypothesized mechanisms are warranted.

Of note, more recently, Furber et al. (40) investigated the relationships between gut microbial communities and athletic performance in a cohort of highly trained individuals underwent dietary periodization (high-carbs vs. high-protein diet). Interestingly, apart from the taxonomic differences between two dietary interventions, the authors revealed that that better athletic performance was linked with gut microbial stasis, where athletes harboring stable microbial communities consistently performed best in each dietary intervention compared to those with a more turbulent gut microbiome.

This result brings to light a pivotal concept: the maintenance of a stable gut microbiome during dietary intervention represents a marker for gut-health and athletic performance (40).

### Differences at phylum level

At phylum level, the decrease in Actinobacteria relative abundance could mainly be attributed to a decrease of the relative abundance of the genus *Bifidobacterium* (Figure 3).

*Bifidobacteria* are common to the healthy human gastrointestinal tract and represent one of the first colonizers of the mammalian gut. *Bifidobacteria* metabolize complex carbohydrates given that the genome of these bacteria harbors many genes involved in carbohydrate metabolism (41, 42). The metagenome includes a variety of genes encoding for a specific hexose fermentation pathway, the fructose-6-phosphate (43), which represent the principal pathway for the energy output produced, compared to classical pathways used by other fermentative intestinal bacteria. Indeed, it provides a growth advantage for *bifidobacteria* in the presence of complex carbohydrates (43). These facts may explain the concomitant proportional decrease of *bifidobacteria* and genes involved in carbohydrate metabolism during KEMEPHY intervention. Accordingly to the reduction in *Bifidobacterium* genus, Turnbaugh et al. (34) recently demonstrated in a cohort of over-weight humans that the drop in bifidobacterial genera was correlated with the increase of ketone bodies and positively associated with a decreased intestinal Th17 cell levels and adipose tissues. Given the links between obesity and chronic low-grade inflammation (44), the authors suggested that decreased levels of pro-inflammatory Th17 cells in both gut and adipose tissues during ketogenic diet may be a potential mechanism contributing to the greater efficacy of ketogenic diet in improving some aspects of metabolic syndrome such as glycemic control (45) and reduction in body fat (46).

A decline in *bifidobacteria* has been also observed in weight loss intervention on a macro nutritionally balanced diet, gluten-free diet and low-gluten intervention diet (47, 48), thus, the reduction of *Bifidobacterium* abundance after KEMEPHY intervention may be also attributed to the low intake of cereal grains.

On the other side, the higher abundance of *Actinobacteria* phylum after WD intervention may be, at least in part, the consequences of the different amount of fibers given that the intake of fibers decreased in the ketogenic diet (from 13 to 11 g per day) while increased in WD diet (from 11 to 15 g per day), which could be a strong driver of *Actinobacteria* abundance (49).

Finally, at phylum level, our analysis also revealed that KEMEPHY intervention altered the composition of the gut microbiome by increasing *Bacteroidetes* and lowering the *Firmicutes* phylum (decreased F:B ratio), compared to WD controls. Even though the F/B ratio is outdated (50), many studies (51–54) have reported that the balance of *Bacteroidetes* and *Firmicutes* may represent an important biomarker for obesity and an indicator of health. More specifically, an increased F:B ratio is commonly associated with dysbiosis, obesity and negative metabolic outcomes (55). These findings are in line with our results since athletes following KEMEPHY underwent a significant reduction in body weight, body fat mass, waist circumference and visceral adipose tissues (13). Moreover, it is well known that an excess of adipose tissue (and particularly visceral adipose tissue, VAT) is related to inflammation (56). In our study, both groups lost body weight, but KEMEPHY group showed a greater reduction of fat mass and VAT.

### Differences and genus level

At genus level, we observed an increased in *Butyricimonas, Clostridia UCG_14, Odoribacter* and *Ruminococcus*. Enrichment of *Butyricimonas* negatively correlated with BMI and triglyceride levels indicates that these taxa may promote health or contribute to the prevention of obesity (57, 58). Our results may support this idea because these taxa increased after KEMEPHY intervention. Moreover, a high abundance of butyric-acid-producing such as *Butyricimonas* has been associated with normal weight and diets high in animal protein and saturated fats (59).

Differently as expected, we observed an increase in the relative abundance of the *Ruminococcus* genus in the KEMEPHY group.

This result is in contrast with previously data which reported an inverse association between *Ruminococcus* abundance and a poly-unsaturated fat-rich diet (60). Indeed, the growth of the genus *Ruminococcus spp*. is usually supported by dietary polysaccharides (3) and individuals consuming animal-based diet or ketogenic diet tend to decrease the levels of the butyrate-producing *Ruminococcus spp*. which are mainly involved in the metabolization of undigested complex dietary carbohydrates and production of SCFAs (3). However, we may speculate that the daily intake of fiber (cellulose, pectin and lignin) provided during KEMEPHY intervention in the food form of fermented foods, berries and vegetables, was adequate to support the growth of *Ruminococcous* bacterial taxa.

Accordingly, we also observed that *Odoribacter* genus increased after KEMEPHY intervention. *Odoribacter*, belonging to the order *Bacteroidales*, is a common SCFAs producing bacteria (61), and, it seems to be associated with some metabolic health benefit such as the improvement of obesity condition (62, 63).

### Importance of up-to-date database

To underline the importance to utilize an up-to-date database in such a new and rapidly growing field as microbiome analysis we reported here, briefly, the most significant differences between our previous analysis performed with Green genes v.13-8 database and the current Silva 138 database. The almost daily advancement in new bacteria classification request the utilization of the most recent database Silva 138. To confirm this fact, the same data showing the main differences are presented in brief and showed in Supplementary Table 1 and Supplementary Figure 2.

### Green genes v.13-8 database vs. Silva 138 database

At phylum level the differences in *Proteobacteria* disappeared with the more recent database, while the phylum of *Actinobacteriota* did not change: it increased in the WD group and decreased in the KEMEPHY group.

At genus level, the main differences were found for *Ruminococcus* and *Dorea* genera. In the previous analysis both genera were slightly reduced in the post condition for KEMEPHY and increased in the WD group, while, with the recent Silva 138 database, the genus of *Ruminococcus* increased in KEMEPHY group while *Dorea* disappeared.

More specifically, Green gene database revealed an increase in *Bifidobacterium, Roseburia, Butyricicoccus* and *Gemmiger* genera in the WD group, and an increase in *Parabacteroides* and *Odoribacterter* genera for KEMEPHY group; differently, the last database revealed an increase in Clostridia UCG-014, *Butyricimonas* and *Odoribacterter* genera in the KEMEPHY group, while the genus of *Paracteroides* disappeared.

### The potential mechanisms of positive effects of KEMEPHY diet on gut microbiome

Our findings suggest that ketogenic diet may partially affect the intestinal ecosystem throughout different mechanisms. We hypothesized that one of these mechanisms might include the production of SCFAs and especially butyrate. Indeed, we supposed that during ketogenic diet, SCFAs and butyrate may be originated from:

i) the liver and then secreted into the gut (because of the ketogenic state);

ii) ketogenic regimens adequately formulated for supplying a medium but adjusted amount of plant-based fermentable fiber to be fermented by SCFAs-producing bacteria;

iii) butyrate producing bacteria such as *Odoribacter, Butyricimonas* and *Ruminococcus*;

iv) specific food sources included in ketogenic diet that may directly provide the adequate amount of butyric acid such as dairy foods (butter and cheese);

v) fermented foods (kefir, yogurt, tempeh), naturally enriched in SCFAs (64–67).

As a matter of fact, butter is one of the richest butyric acid food sources with an inherent natural supply of 3–4% of fat content as butyric acid. For example, one tablespoon of butter is composed of 560 mg of butyric acid (68). Thus, for individuals following a ketogenic diet, it is easily possible to consume well more than 1,000 mg of butyrate in a day, from natural sources (68). Hence, butyrate acts in synergy with the ketogenic goals since it represents a direct substrate to undergo beta-oxidation (69).

In line with these concepts, Nagpal et al. observed a slight increase in fecal butyrate after 6-weeks of modified Mediterranean-ketogenic diet. The authors supposed that the butyrate might have originated in the liver as consequence of the ketogenic state, or the ketogenic diet might have promoted the intestinal production of butyrate by supplying plant-based fermentable fibers to be fermented by bacteria (65).

Notably, it should be also underlined that our KEMEPHY was composed also of functional fermented products (kefir, kimchi, whole yogurt and fermented cheese) which are naturally enriched in short-chain fatty acids (64).

In addition, beta-hydroxybutyrate derived from hepatic production during ketogenesis, has also the ability to influence, directly or indirectly, the gut microbiome, providing additional support for the fundamental function of ketone bodies at both intestinal and systemic level (34).

### Current limitations

Despite these interesting results, our study is not without limitations. First, the reduced sample size of our cohort of athletes may represent a limit for a real robust statistical difference in gut microbiome profiling. Moreover, our analysis has been performed with 16S rRNA gene sequencing which represent the most applied method to investigating gut microbiome, but it is not efficient as shotgun metagenomic sequencing (70). Indeed, 16s rRNA targets and reads a region of the 16S rRNA gene while shotgun technique sequences all given genomic DNA while achieving strain-level resolution. The results is that 16S rRNA gene sequencing detects only part of the gut microbiome community revealed by shotgun sequencing and it does not provide a functional profiling of gut microbes (71). However, a technical challenge was considerable at the time of analysis. Since our research was conducted there years ago and shotgun metagenomic was orders of magnitude more expensive and relatively new than amplicon analysis (~$150 USD for shotgun and ~$50 USD for 16S), at that moment, 16S rRNA sequencing represented the best and most used method for microbiome studies. Moreover, it is important to highlight that also regular physical exercise, such as that performed by our cohort of semi-professional soccer players, might have influenced the results of the study by promoting the maintenance of a functional and physiological microbiota in both groups (72).

Further studies on KD on athletes would help validate these findings in gut microbiome and, thanks to the innovative available bioinformatic platforms, the integration of omics-data with the metagenomic methods may improve the understanding of the relationship between diet, gut microbiome and physical exercise (73). In addition, our study did not measure the level of SCFAs that could be an additional finding helping the explanation of the underlying mechanisms and of the interpretation of results.

## Conclusion

There is a growing body of research on the role of gut microbiome in sport and performance. For the first time our results demonstrate that (i) KEMEPHY diet may be considered a feasible and safe nutritional strategy for athletes to get an adequate body composition, (ii) KEMEPHY diet do not change the overall composition of gut microbiome and, (iii) 30 days of KEMEPHY intervention may represent an alternative tool for maintaining and/or modulating the composition of gut microbiome in athletes practicing regular exercise. These findings suggest that KEMEPHY diet may represent an efficient dietary pattern for athletes, according to the notion that preserving a stable gut microbiome during dietary intervention represent a marker of gut health and greater athletic performance.

It should be stressed that our KEMEPHY diet was mainly composed by healthy fats (good sources of monounsaturated and polyunsaturated fats), fibers (low-carb veggies, seeds), plant-based protein (tofu, tempeh) and fermented foods (kefir, tempeh, yogurt, kimchi), different from a standard high fat–low fibers ketogenic diet, which may not arouse the same beneficial effects on gut microbiome. Our findings demonstrate also that changes in microbial taxa pre and post intervention significantly correlate with environmental variables such as athlete's macronutrient intake.

Finally, it should be emphasized that data analysis performed with not updated database may give back partially different results as we demonstrated here.

## Data availability statement

The data presented in the study are deposited in the NCBI SRA repository, accession number PRJNA865651.

## Ethics statement

The studies involving human participants were reviewed and approved by Ethical Committee of the Department of Biomedical Sciences, University of Padua. The patients/participants provided their written informed consent to participate in this study.

## Author contributions

AP and LM conceived and drawn the experiment and wrote the manuscript. LM collected the gut microbiota data. LB and ES performed the 16S rRNA sequencing analysis. LM, SA, and DS performed the statistical analysis of microbiome data. AP, LM, and LC designed the nutritional protocols. AB contribute to the discussion. MC revised critically the manuscript. All authors read and approved the final manuscript.

## Funding

This work was funded by the Department of Biomedical Sciences, University of Padua Institutional Grant.

## Conflict of interest

Author AP has received a research grant from Gianluca Mech S.p.A. Asigliano Veneto, Vicenza, Italy. Author LC is a Ph.D. student currently supported by a grant from Gianluca Mech SpA, Asigliano Veneto, Vicenza, Italy. The company had no role in the study design, data collection, data analysis, data interpretation, or writing of the article. Authors ES and LB were employed by BMR Genomics srl.

The remaining authors declare that the research was conducted in the absence of any commercial or financial relationships that could be construed as a potential conflict of interest.

## Publisher's note

All claims expressed in this article are solely those of the authors and do not necessarily represent those of their affiliated organizations, or those of the publisher, the editors and the reviewers. Any product that may be evaluated in this article, or claim that may be made by its manufacturer, is not guaranteed or endorsed by the publisher.

## Supplementary material

Supplementary Figure 1 Rarefaction plot. All the samples included in the analysis was rarefied. The value of rarefaction is 32232 reads.

Supplementary Figure 2 Relative abundance (in log 10 scale) of the more represented phyla (>0.1%) in the pre- and post-intervention, analyzed with Green Genes database. Stars represent a significant time × group interaction (*p* < 0.05).

Supplementary Table 1 Relative abundance of the more represented phyla (>0.1%) in the pre- and post-intervention, for KDP and WD groups, analyzed with Green Genes database. Stars represent a significant time × group interaction (*p* < 0.05).
