# Supplementary figures and images for: Effect of 30 days of ketogenic Mediterranean diet with phytoextracts on athletes' gut microbiome composition
Source: Front Nutr. 2022 Oct 25;9:979651. doi: 10.3389/fnut.2022.979651 (PMC9642348; doi:10.3389/fnut.2022.979651)

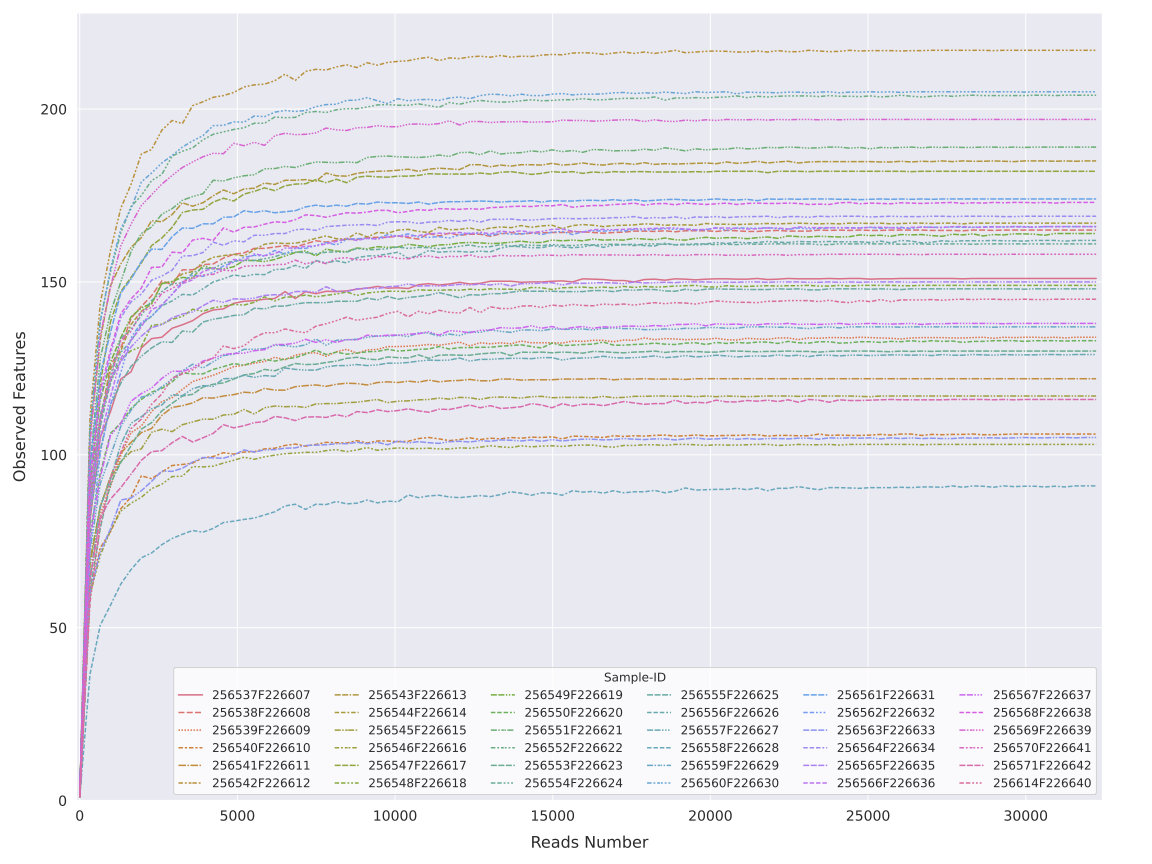

Supplement: Supplementary Figure 1 — Rarefaction plot. All the samples included in the analysis was rarefied. The value of rarefaction is 32232 reads. [file Image_1.TIFF]

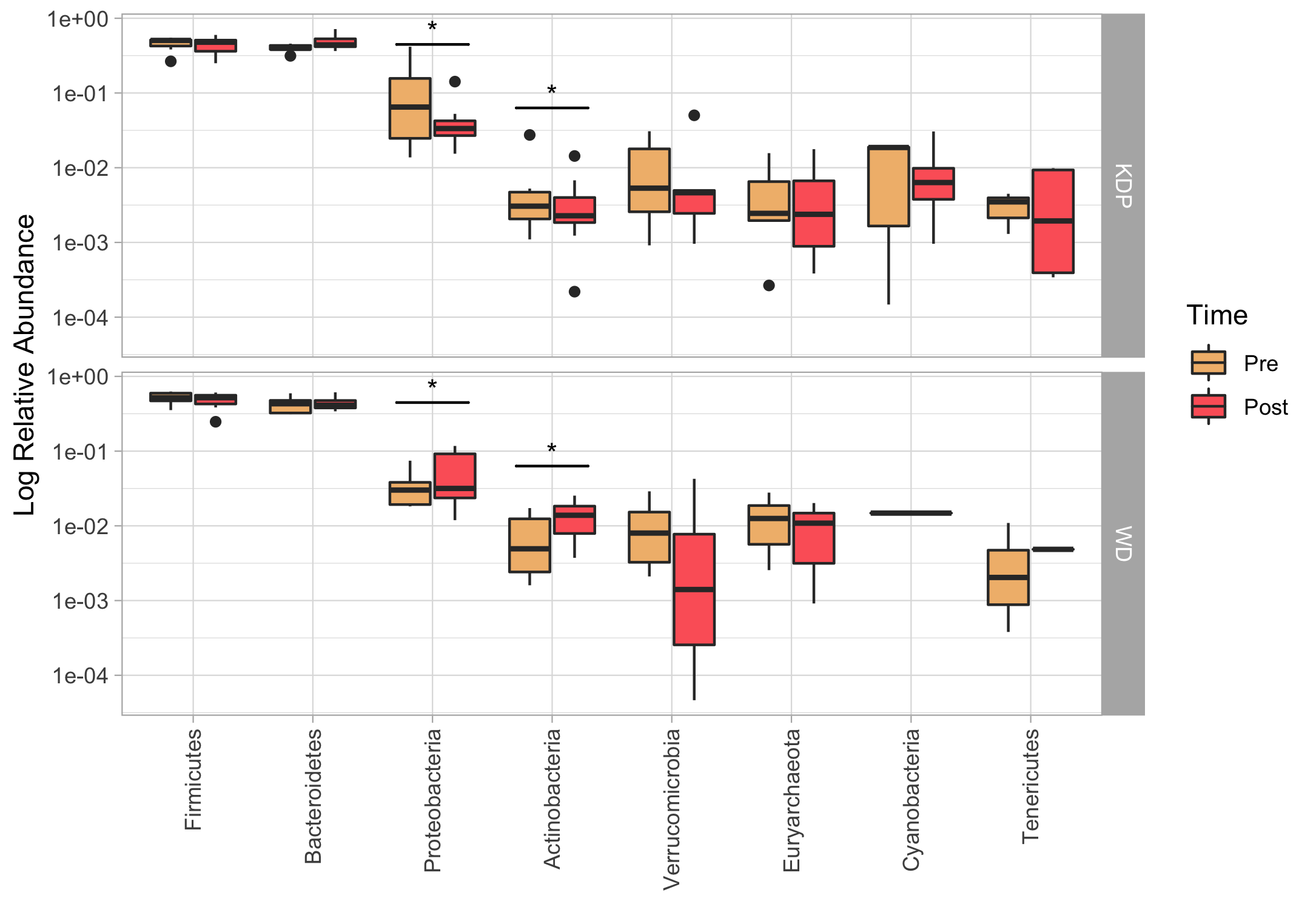

Supplement: Supplementary Figure 2 — Relative abundance (in log 10 scale) of the more represented phyla (>0.1%) in the pre- and post-intervention, analyzed with Green Genes database. Stars represent a significant time × group interaction (p < 0.05). [file Image_2.TIFF]
