# Supplementary material for: Effect of 30 days of ketogenic Mediterranean diet with phytoextracts on athletes' gut microbiome composition
Source: Front Nutr. 2022 Oct 25;9:979651. doi: 10.3389/fnut.2022.979651 (PMC9642348; doi:10.3389/fnut.2022.979651)
Supplement: Supplementary Table 1 — Relative abundance of the more represented phyla (>0.1%) in the pre- and post-intervention, for KDP and WD groups, analyzed with Green Genes database. Stars represent a significant time × group interaction (p < 0.05). [file Table_1.docx]

**Supplemental Table 1:**

Relative abundance of the more represented phyla (>0.1%) in the pre- and

post-intervention, for KDP and WD groups, analyzed with Green Genes database. Stars represent a significant time×group interaction (p<0.05).

|  | **KDP** | | **WD** | |  |  |  |
| --- | --- | --- | --- | --- | --- | --- | --- |
| **Phylum** | **Pre** | **Post** | **Pre** | **Post** | **Time** | **Group** | **Time* Group** |
| *Actinobacteria* | 0.3 (0.2-0.5) | 0.2 (0.2-0.4) | 0.5 (0.2-1.2) | 1.4 (0.8-1.9) | 0.274 | 0.014 | 0.021* |
| *Bacteroidetes* | 39.6 (38.3-43.3) | 44.0 (41.7-53.4) | 43.0 (32.4-47.8) | 41.2 (37.9-47.6) | 0.058 | 0.696 | 0.161 |
| *Cyanobacteria* | 0.0 (0.0-0.5) | 0.0 (0.0-0.6) | 0.0 (0.0-0.0) | 0.0 (0.0-0.0) | 0.787 | 0.020 | 0.180 |
| *Euryarchaeota* | 0.1 (0.0-0.3) | 0.0 (0.2-0.3) | 0.0 (0.0-0.5) | 0.0 (0.0-0.3) | 0.529 | 0.638 | 0.911 |
| *Firmicutes* | 50.5 (42.6-51.3) | 47.8 (36.2-50.9) | 51.9 (47.1-59.6) | 51.9 (42.9-56.0) | 0.093 | 0.184 | 0.878 |
| *Fusobacteria* | 0.0 (0.0-0.0) | 0.0 (0.0-0.0) | 0.0 (0.0-0.0) | 0.0 (0.0-0.0) | 1.000 | 0.349 | 0.382 |
| *Proteobacteria* | 6.5 (2.5-15.7) | 3.4 (2.7-4.3) | 3.0 (1.9-3.8) | 3.2 (2.4-9.2) | 0.528 | 0.361 | 0.028* |
| *Spirochaetes* | 0.0 (0.0-0.0) | 0.0 (0.0-0.1) | 0.0 (0.0-0.0) | 0.0 (0.0-0.0) | 0.059 | 0.612 | 0.566 |
| *Tenericutes* | 0.0 (0.0-0.2) | 0.0 (0.0-0.3) | 0.0 (0.0-0.0) | 0.0 (0.0-0.0) | 0.834 | 0.184 | 0.131 |
| *Verrucomicrobia* | 0.3 (0.1-1.2) | 0.2 (0.0-0.5) | 0.3 (0.0-1.0) | 0.0 (0.0-0.0) | 0.351 | 0.339 | 0.957 |
| *Unknown* | 0.0 (0.0-0.1) | 0.0 (0.0-0.0) | 0.0 (0.0-0.1) | 0.0 (0.0-0.0) | 0.744 | 0.880 | 0.798 |

Stars represent the values that are statistically significant.
